# Clinical Applications of Molecular Biomarkers in Prostate Cancer

**DOI:** 10.3390/cancers12061550

**Published:** 2020-06-12

**Authors:** Felipe Couñago, Fernando López-Campos, Ana Aurora Díaz-Gavela, Elena Almagro, Esaú Fenández-Pascual, Iván Henríquez, Rebeca Lozano, Estefanía Linares Espinós, Alfonso Gómez-Iturriaga, Guillermo de Velasco, Luis Miguel Quintana Franco, Ignacio Rodríguez-Melcón, José López-Torrecilla, Daniel E. Spratt, Luis Leonardo Guerrero, Juan Ignacio Martínez-Salamanca, Elia del Cerro

**Affiliations:** 1 Radiation Oncology, Hospital Universitario Quirónsalud Madrid, 28223 Madrid, Spain; adiazg@quironsalud.es (A.A.D.-G.); leoguerrero01@gmail.com (L.L.G.); elia.delcerro@gmail.com (E.d.C.); 2 Radiation Oncology, Hospital La Luz, 28003 Madrid, Spain; 3 Clinical Department, Faculty of Biomedicine. Universidad Europea de Madrid, 28670 Madrid, Spain; 4 Radiation Oncology, Hospital Universitario Ramón y Cajal, 28034 Madrid, Spain; fernando_lopez_campos@hotmail.com; 5 Medical Oncology, Hospital Universitario Quirónsalud Madrid, 28223 Madrid, Spain; e.almagro.c@gmail.com; 6 Lyx Institute of Urology, Universidad Francisco de Vitoria, 28006 Madrid, Spain; esau.fdez@gmail.com (E.F.-P.); estefanialinares@gmail.com (E.L.E.); 7 Department of Urology, Hospital Universitario La Paz, 28046 Madrid, Spain; luismi.quintanafranco@gmail.com; 8 Radiation Oncology, Hospital Universitario Sant Joan, 43204 Reus, Spain; ivanhenriquezlopez@me.com; 9 Prostate Cancer Clinical Research Unit, Spanish National Cancer Research Centre, 28029 Madrid, Spain; rlozano@ext.cnio.es; 10 Genitourinary Cancer Traslational Research Group, Institute of Biomedical Research, 29010 Málaga, Spain; 11 Cruces University Hospital, Biocruces Heath Research Institute, 48903 Barakaldo, Spain; agomeziturriaga@gmail.com; 12 Medical Oncology, Hospital Universitario 12 de Octubre, 28041 Madrid, Spain; gdvelasco.gdv@gmail.com; 13 Radiation Oncology, Hospital Universitario de Gran Canaria Dr. Negrín, 35010 Las Palmas de Gran Canaria, Spain; nachorodriguezmelcon@hotmail.com; 14 Radiation Oncology-ERESA, Hospital General Universitario de Valencia, 46014 Valencia, Spain; jltorrecilla@eresa.com; 15 Department of Radiation Oncology, University of Michigan, Ann Arbor, MI 48109, USA; sprattda@med.umich.edu; 16 Department of Urology, Hospital Universitario Puerta de Hierro, 28222 Madrid, Spain

**Keywords:** biomarkers, DNA repair, gene panels, genetic testing, germline mutation, precision medicine, prostate cancer, prostate genomics

## Abstract

There is clinically relevant molecular heterogeneity in prostate cancer (PCa), but this biological diversity has had only a minimal impact on clinical practice. Treatment outcomes in patients with localised PCa are often highly variable, even among patients stratified to the same risk group or disease state based on standard clinical and pathological parameters. In recent years, the development of gene panels has provided valuable data on the differential expression of genes in patients with PCa. Nevertheless, there is an urgent need to identify and validate prognostic and predictive biomarkers that can be applied across clinical scenarios, ranging from localised disease to metastatic castration-resistant PCa. The availability of such tools would allow for precision medicine to finally reach PCa patients. In this review, we evaluate current data on molecular biomarkers for PCa, with an emphasis on the biomarkers and gene panels with the most robust evidence to support their application in routine clinical practice.

## 1. Introduction

Prostate cancer (PCa) is the most common cancer in men—accounting for 19% of all cancers in males—and the second leading cause of cancer-specific mortality in this population [1]. The introduction of prostate-specific antigen (PSA) testing and screening in the late 1980s doubled the incidence of PCa, which in turn reduced PCa-specific mortality rates [2,3,4]. However, routine PSA testing led to overdiagnosis and perhaps also overtreatment, particularly prostate biopsies [5]. The widespread use of diagnostic biopsies—a highly invasive procedure associated with a non-negligible increase in morbidity and mortality—has increased the diagnosis of clinically-insignificant PCa [6]. Given these circumstances, the main question surrounding the optimal management of PCa is not the selection of the most appropriate active treatment, but rather the diagnostic process itself, particularly due to the low specificity of PSA tests. Consequently, new biomarkers are needed to better optimise the diagnosis of PCa, which would help to avoid unnecessary biopsies while simultaneously increasing the probability of a positive biopsy. In turn, this would increase the proportion of biopsied patients diagnosed with clinically-significant PCa.

Once a patient has been diagnosed with PCa, their disease is commonly classified according to clinical and pathological criteria (e.g., the National Comprehensive Cancer Network (NCCN), D’Amico, AJCC Cancer Staging) [7,8,9], which stratify patients into risk groups. These systems are largely based on intrinsic characteristics of the tumour (e.g., Gleason score; GS), clinical parameters such as tumour stage (TNM), and pre-treatment prostate-specific antigen (PSA) values. Although these stratification systems provide important prognostic information about the expected behaviour of the tumour, the clinical reality is that the performance of these prognostic systems is suboptimal in discriminating biologically aggressive tumours. Thus, some cancers currently classified as “intermediate” risk are actually more aggressive than “high” risk tumors, thus leading to both over- and under-treatment.

In addition to changes pertaining to diagnosis and prognosis, true personalised medicine guided by predictive biomarkers is largely absent in the current clinical management of PCa. At present, the optimal application and combination of the available treatments—active surveillance, surgery, radiotherapy, androgen-deprivation therapy (ADT), next-generation androgen receptor signalling inhibitors (ARSI), chemotherapy, immunotherapy, etc.—is not clear. Given the complexity of therapeutic decision-making in these patients, we need to determine which patients are most likely to benefit from a given treatment, establish the optimal sequence of treatments, and enrol appropriate and selected patients in clinical trials involving targeted therapies [10].

In this context, the aim of the present review is to assess the latest scientific evidence to determine the current role and limitations of molecular biomarkers and gene panels in different clinical scenarios, including from localised, recurrent, non-metastatic castration resistant (nmCRPC), metastatic hormone sensitive (mHSPC), and metastatic castration-resistant disease (mCRPC). We also discuss the potential impact of these tests on clinical decision-making and future lines of development.

## 2. Risk Assessment Biomarkers

### 2.1. Serum Markers

#### 2.1.1. Prostate Health Index (PHI)

The PHI combines total PSA, fPSA (free non-protein bound PSA), and p2PSA (an isoform of fPSA resulting from incomplete processing of the PSA precursor). The PHI score is calculated according to the following formula: (2pPSA/fPSA) × √PSA, which implies that men with a higher total PSA and p2PSA and a lower fPSA have a higher risk of presenting clinically-significant PCa [11]. The PHI has been shown to improve the area under the curve (AUC) versus PSA for the detection of all prostate cancers, regardless of risk group (AUC: PHI = 0.708 vs. PSA = 0.516) and for the detection of high-grade PCa (Gleason ≥7): AUC: PHI = 0.707 vs. PSA = 0.551 [12]. At a PHI cut-off value ≥25, 40% of biopsies would not be performed and the diagnosis of cases of lower grade PCa (GS = 6) would be reduced by 25%; however, the trade-off is that approximately 5% of clinically-significant cases would be missed [13].

#### 2.1.2. KScore

The 4Kscore incorporates both serum biomarkers (tPSA, fPSA, intact PSA, and human kallikrein 2) and clinical variables (age, digital rectal examination (DRE), and previous biopsy results) to predict the risk of high-grade PCa on the biopsy. This score has been validated for both initial and repeat biopsies [14]. A systematic review assessed the performance of the 4Kscore in the pre-biopsy setting, reporting a pooled AUC > 0.80 for the detection of clinically-significant prostate cancer (csPCa); importantly, that finding was highly consistent across the 11 studies (>10,000 patients) included in the review [15]. On average, using a 4K score cut-off of 9% to indicate a systematic biopsy could prevent 43% of biopsies, but at the cost of missing 2.4% of cases with csPCa [16,17].

Nordström et al. compared the PHI to the 4Kscore in 531 men who underwent an initial biopsy, finding that the two tests had a similar capacity to predict csPCa (AUC 0.71 vs. 0.72, respectively) [18]. Based on those findings, the authors concluded that these two serum biomarkers (PHI and 4Kscore) offer comparable performance.

#### 2.1.3. Other Blood Based Biomarkers

The Stockholm 3 Model (S3M), based on data from the study of the same name, combines numerous serum biomarkers (PSA, free PSA, hK2, MSMB, MIC1, HOXB13), genetic polymorphisms (232 single-nucleotide polymorphisms), and clinical parameters (age, family history, previous prostate biopsy, and DRE). The S3M has been proposed to determine the indications for an initial biopsy. In the STHLM3 cohort, this model significantly outperformed all of the individual parameters in detecting csPCa (AUC 0.76) [19].

### 2.2. Urinary Markers

#### 2.2.1. Prostate Cancer Antigen 3 (PCA3)

PCA3 is a gene that transcribes a long, noncoding messenger RNA (mRNA) that is overexpressed in PCa tissue and detectable in urine after DRE. The PCA3 score is calculated by measuring the concentration of PCA3 mRNA relative to PSA mRNA [20]. Although this marker has not been associated with csPCa, it has been shown to improve the sensitivity and specificity of second biopsies to detect all grades of PCa, with repeat biopsy indicated in patients with scores >35 [21]. Comparative studies have shown that the PHI is superior to the PCA3 as a predictor of csPCa on biopsy [22].

#### 2.2.2. TMPRSS2-ERG Fusion Gene

The genomic rearrangement of TMPRSS2-ERG, attributable to the translocation of the TMPRSS2 chromosomal region and the ERG transcription factor belonging to the ETS family of transcription factors [23,24,25]. TMPRSS2 is an androgen-regulated gene, located 3Mb apart on the same 21q22 chromosome. ERG is overexpressed in 40%–50% of primary prostate tumours [26], with ETS gene rearrangements—mainly TMPRSS2-ERG fusion (>90% ETS rearrangements)—described in 30%–50% of patients with newly-diagnosed, untreated PCa [27].

For diagnostic purposes, the TMPRSS2-ERG fusion gene can be detected in post-DRE urine samples, with a specificity of 93% and a positive predictive value (PPV) of 94% for the diagnosis of PCa, which is independent of the PCA3 value [28].

#### 2.2.3. MiProstate Score (MiPS)

The combination of PCA3 with TMPRSS2-ERG as a risk estimation model provides additional information to help guide decision-making for prostate biopsy. MiPS is also a promising biomarker to estimate the risk of a diagnosis of PCa [29]. The AUC of the MiPS is 0.88, with a sensitivity and specificity of 80% and 90%, respectively, for the detection of PCa, and an AUC of 0.772 for csPCa. The MiPS, which is based on post-DRE urine samples, can help to avoid unnecessary biopsies when used in combination with total PSA [30].

#### 2.2.4. SelectMDx

SelectMDx is a test designed to measure RNA levels of two genes (*DLX1* and *HOXC6*) in post-DRE urine samples. The algorithm used to calculate the SelectMDx score includes tPSA, PSA density, DRE, age, and family history, which are combined to estimate the probability of a diagnosis of PCa (all grades) and csPCa. This tool was developed by Van Neste et al. [31] based on data from 519 men who underwent prostate biopsy and later validated in a cohort of 386 patients. SelectMDx was developed to improve the selection of candidates for initial prostate biopsy. The AUC value for csPCa is 0.90, with a negative predictive value (NPV) of 98%, which is superior to the base model without RNA markers and also better than the PCa prevention trial (PCPT) risk calculator. A decision curve analysis suggested that application of SelectMDx could prevent 42% of biopsies while only missing 2% of cases with csPCa [31].

#### 2.2.5. ExoDx Prostate Intelliscore

**Figure 1:**
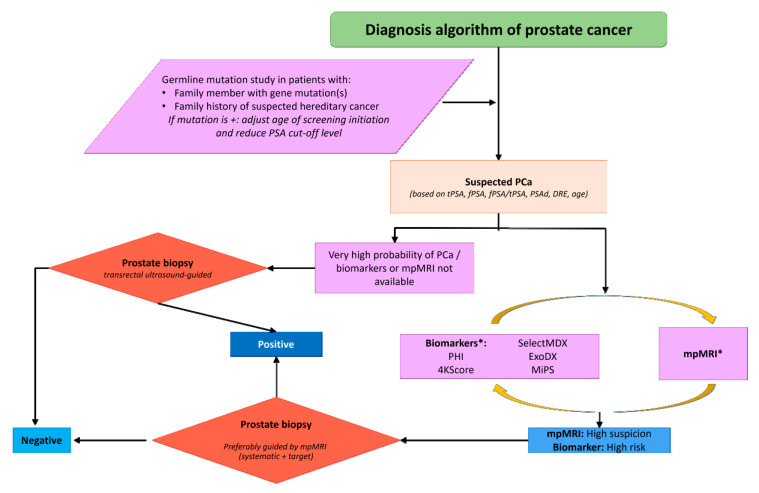
Diagnostic algorithm for prostate cancer patients. PSA: prostate-specific antigen; PCa: prostate cancer; tPSA: total serum PSA; fPSA: free non-protein bound PSA; PSAd: PSA density; DRE: digital rectal examination; mpMRI: multiparametric magnetic resonance imaging; PHI: Prostate Health Index; MiPS: MiProstate Score.

Exosomes and endosomes are small extracellular vesicles (EVs) (30–100 nm in size) involved in intercellular communication. These nanovesicles have generated much interest as potential biomarkers due to their stability—the content within the EV is protected from enzymatic degradation by the EV lipid bilayer [32]—and because they are easy to detect in different fluids (blood, urine, or semen) [33]. EVs contain molecules such as proteins, DNA, mRNA, or miRNA, and their function is to transport these molecules from one cell to another, exchanging genetic information, and/or reprogramming the recipient cells. There is a growing body of evidence suggesting that tumour cells release a large number of EVs potentially associated with tumour growth, progression, and/or treatment resistance [34]. Recent data have shown that two biomarkers (TMPRSS2-ERG and PCA3) can be detected in EVs isolated from urine samples obtained from patients with PCa [35]. The EXO106 score is based on the analysis of RNA content in EVs isolated from the urine of men with suspected PCa, calculated as the sum of normalised PCA3 and EV RNA ERG. The reported NPV and PPV values for the EXO106 to detect csPCa are 97.5% and 34.5%, respectively [36]. The combination of the EXO106 score with clinical parameters (PSA, age, race, and family history) improves discrimination between clinically-significant and non-significant PCa compared to standard approaches [37]. This determination, based on the analysis of EVs in urine without prior DRE, demonstrated good accuracy (AUC = 0.73), leading to the development of the ExosomeDx test (ExoDx Prostate Intelliscore), of which the aim is to reduce unnecessary biopsies in patients with or without previous biopsy [36,38] (Figure 1).

### 2.3. Tissue Markers: ConfirmMDx

The ConfirmMDx (MDxHealth) is a tissue-based gene assay for epigenetic testing. This panel is based on methylation studies and was developed to assess the prognostic value of specific epigenetic alterations in PCa. The results of this assay can provide useful data to improve decision-making to indicate the value of a repeat biopsy after an initial negative result. The assay analyses the hypermethylation of three biomarkers (GSTP1, APC, and RASSF1) in the tissue obtained from a negative biopsy [39], with a 90% NPV at 30 months after the initial biopsy. These results were validated in another study, which concluded that ConfirmMDx is a significant independent predictor for prostate biopsy outcomes [40].

### 2.4. Imaging Tests: Multiparametric Magnetic Resonance Imaging (mpMRI)

Magnetic resonance imaging (mpMRI) is the imaging test of choice in patients suspected of presenting csPCa [41]. Multiparametric MRI allows clinicians to perform a focal biopsy, typically in combination with a systematic biopsy. The PI-RADS v2.1 system uses a five-point scale (1–5) to assess the probability that a tumour is clinically significant, with scores of four or five considered suspicious [42]. Studies have investigated the use of mpMRI findings as an “imaging biomarker” in combination with other biomarkers.

The PRECISION and MRI-FIRST studies evaluated the utility of mpMRI prior to the first prostate biopsy [43,44]. A Cochrane review that assessed more than 5000 patients concluded that prebiopsy mpMRI reduces the overdetection of 37% of cases of clinically-insignificant cases of PCa, reducing biopsies by 32%, although at the cost of missing 4% of cases with csPCa [45].

In patients presenting a high degree of suspicion of PCa despite a negative biopsy, the guidelines of the European Association of Urology (EAU) and the National Comprehensive Cancer Network (NCCN) recommend performing mpMRI as this technique detects 44% more cases of csPCa, avoids 38% of non-significant cases, and reduces the number of biopsies by 32%, while only missing approximately 2% of clinically-significant cases [14,15,16,17,18,19,20,21,22,23,24,25,26,27,28,29,30,31,32,33,34,35,36,37,38,39,40,41,45].

Given the extra cost of prebiopsy mpMRI, some studies have evaluated the utility of determining the 4KScore or PCA3 before proceeding to mpMRI [46,47]. The use of SelectMDX after mpMRI to detect patients with PI-RADS 3 considered candidates for biopsy has also been evaluated. The results of these diagnostic algorithms (and each individual biomarker) are described in Table 1 [48].

Although all the biomarkers described in Table 1 outperform the PSA as a diagnostic tool, their routine use in clinical practice is still limited, partly due to their limitations. The aim of using these biomarkers is to reduce the number of unnecessary biopsies and to better discriminate csPCa from ncsPCa to avoid overdiagnosis. The development of gene panels that combine different biomarkers together with clinical and radiological parameters appears to be the alternative that most significantly improves the diagnosis of PCa. In this regard, there is a clear and urgent need to validate and prospectively compare these markers with each other.

## 3. Biomarkers of Susceptibility

Germline mutations in BRCA1 and BRCA2 are associated with an increased risk of developing several types of cancer, including breast, prostate, and ovarian cancer. When a germline mutation in BRCA1 or BRCA2 is detected in a cancer patient, genetic testing is indicated in all family members due to the increased risk associated with these mutations. Nevertheless, studies conducted in patients with PCa [49,50,51] have found that more than 30% of patients with germline mutations in DNA repair genes (and 15% of those with the BRCA2 mutation) do not have any family members diagnosed with cancer.

Published data suggest that genetic factors account for up to 57% of interindividual variation in the risk of developing PCa [52]. Men with the BRCA2 germline mutation have a 30% lifetime risk of developing PCa, although this risk varies greatly (from 19% to 61%) according to the presence/absence of genetic variants, which act as risk modifiers [53]. The main genetic factor associated with a higher risk of developing PCa is a germline mutation in BRCA2, with a relative risk (RR) of 8.6 in males under age 65 [54]. BRCA1 mutations are also associated with an increased risk of PCa, although the effect is less pronounced, with a RR of 4.5 in males under age 65 [55]. Germline mutations in HOXB13 have also been associated with an increased risk of PCa and targeted genomic analysis of this gene could help to identify families at high risk [56]

There is a growing body of evidence demonstrating the clinical relevance of germline and somatic mutations in DNA damage repair genes. BRCA2 mutations are associated with a more aggressive disease course and worse clinical outcomes [57,58,59,60,61]. The clinical significance of BRCA1 and BRCA2 germline mutations has been demonstrated in several studies [62]. Although the sample sizes in those studies were small, the findings suggest that the presence of a BRCA mutation—even among patients with similar PSA values at diagnosis—is associated with a greater likelihood of nodal involvement, distant metastases, and Gleason score ≥ 8 at diagnosis when compared to patients without these genomic aberrations [60]. Consequently, BRCA2 germline mutations are a prognostic factor—independent of other classical prognostic factors such as PSA level at diagnosis or tumour stage (TNM)—for the development of metastasis and survival outcomes. In fact, the BRCA2 germline mutation was the first genetic factor proven to impact the prognosis of PCa. Accordingly, the most recent National Cancer Comprehensive Network (NCCN) guidelines recommend screening for germline mutations in DNA damage repair (DDR) genes in all patients with metastatic, regional, high-risk, or very high-risk disease, regardless of family history [7].

The presence of germline mutations in patients with PCa has implications not only for the patient, but for all family members, who should also undergo genetic testing to ensure early detection of cancer and, if appropriate, the implementation of risk reduction strategies in mutation carriers [63].

International guidelines recommend annual PSA screening for PCa in the general population between 40–45 years of age [64]. The IMPACT study evaluated the efficacy of this screening approach in patients with BRCA germline mutations [65]. That study found that patients with the BRCA2 mutation were diagnosed at a younger age and also more likely to present clinically-significant disease at diagnosis (intermediate or high-risk PCa in 77% of BRCA2 carriers versus 40% in controls). These findings underscore the need to optimise early detection strategies in men with a high genetic risk—particularly germline mutations in BRCA2—with early screening performed before age 45 or, in patients with a family member diagnosed with cancer, 10 years before the age of diagnosis. In addition, the PSA threshold in these patients should be lowered to 2.5 ng/dL and other biomarkers should be assessed as part of the diagnostic process [66].

In summary, an individual’s predisposition to develop PCa is partly determined by genetic susceptibility. Although the clinical implications of germline genetic mutations in DDR genes are yet to be elucidated, the impact of inherited BRCA2 mutations has been well characterised and will allow, in the near future, the development of anticipatory strategies, targeted screening programs, and personalised treatment.

## 4. Molecular Biomarkers in Localised Prostate Cancer

In recent years, the management of localised PCa has undergone a paradigm shift, starting with the premise that localised disease is potentially curable. It is essential to use all available tools to identify the patients most likely to benefit from a given intervention or, if appropriate, to closely monitor patients with indolent tumours. In addition, it is important to determine whether the patient with localised PCa is a candidate for adjuvant radiotherapy (ART) or salvage radiotherapy (SRT), the indication for hormonotherapy (HT) after prostatectomy, and to quantify the impact of HT in non-surgical intermediate-risk patients.

Several genetic tests have been validated for different clinical scenarios in recent years. These tests can improve prognostic estimates for the likelihood that the prostatectomy specimen will present unfavourable pathological findings and can also estimate the probability of biochemical control and metastasis-free survival. Note, however, that current biomarkers and molecular assays are based on data from patients who underwent active treatment (radiotherapy and/or prostatectomy). Consequently, the results of these tests in untreated patients must be interpreted cautiously, and any prognostic estimate based on those results should be considered carefully.

Numerous gene panels have been developed for localised PCa, although only four are commercially available at present: ProMark, Prolaris, Oncotype Dx Prostate and Decipher. All of these tests—despite variability in the methodological quality of the validation studies—can predict the risk of clinically-significant disease to better characterise patients and improve therapeutic decision-making.

### 4.1. ProMark

ProMark^®^ (Metamark Genetics Inc., Waltham, MA, USA) is a gene profile assay that analyses the expression of eight different protein markers. ProMark scores range from zero to one to indicate the probability of detecting adverse pathology in the radical prostatectomy specimen. Patients are classified into risk groups that provide independent prognostic data based on the initial prostate biopsy. ProMark was developed to reduce inconsistencies related to improper biopsy techniques and subjectivity in grading tumour aggressiveness (as this is pathologist-dependent) [67]. Higher risk scores on this biomarker are correlated with a lower likelihood of favourable pathologic characteristics: for scores >0.8, the predictive value for unfavourable pathologic characteristics after prostatectomy can be as high as 76.9% [68].

### 4.2. Prolaris

The Prolaris test (Myriad Genetics, Salt Lake City, UT), which was developed in 2010, is one of the most widely used gene panels in PCa. Prolaris is based on the determination and combination of expression levels of 31 genes involved in cell cycle progression and 15 housekeeper genes. This test has been validated in four different studies, which have demonstrated that Prolaris adds prognostic value to traditional clinical models (i.e., risk groups) and to Ki-67 values; importantly, this test was developed in a reference laboratory and is easily reproducible [69]. The Prolaris test yields a proliferation index expressed as the cell cycle progression (CCP) score, which measures the aggressiveness of PCa.

The first validation study involved tissue specimens obtained via transrectal/transperineal needle biopsy in a subgroup of untreated patients to determine the probability of PCa-specific mortality [70]. Two subsequent studies confirmed the value of the test as a prognostic factor for biochemical recurrence and metastatic progression in patients treated with prostatectomy [71]. However, those conclusions were based on analysis of two different tissues—one study analysed the prostatectomy specimen while the other analysed the pre-prostatectomy biopsy specimen [69,72,73]. After prostatectomy, each unit increase in the Prolaris score was associated with a doubling of the risk of biochemical recurrence; the score was also shown to predict mortality after progression [72]. Freedland et al. found that the Prolaris score was correlated with biochemical recurrence and disease-free survival in patients treated with external radiotherapy [73]. Another study assessed the potential impact of the Prolaris test on routine clinical practice, concluding that the results of this genetic test would have altered the original treatment recommendation in 65% of cases [74,75].

### 4.3. Oncotype Dx

In 2014, Cooperberg et al. described a new gene panel developed for men with intermediate or favourable risk PCa known as the Oncotype DX Genomic Prostate Score (Genomic Health, Redwood City, CA, USA). Oncotype Dx is based on quantitative RT-PCR tumour analysis of 12 genes associated with PCa and five internal reference genes across four molecular pathways: androgen signalling, stromal response, cellular organisation, and cellular proliferation. The combined expression of these genes evaluated through an established algorithm yields the Genomic Prostate Score (GPS), which was initially validated by Klein et al., in prostatectomy specimens obtained from 395 patients with low and intermediate-risk disease. Those authors found that the GPS was significantly associated with tumour grade and pathological stage (OR: 2.1; 95% CI: 1.4–3.2; *p* < 0.001). Moreover, combining the GPS with the CAPRA (Cancer of the Prostate Risk Assessment) score improved the AUC for favourable pathological findings versus the CAPRA score alone. Based on those findings, the authors concluded that the Oncotype Dx test predicts the risk of more unfavourable PCa on histological analysis or a higher T stage after prostatectomy. In practical terms, this test can be applied to determine if a candidate for active surveillance could benefit from radical treatment [76,77]. However, the first multicentre prospective study evaluating the use of GPS after initial active surveillance has recently been published, without demonstrating an independent association of GPS with adverse pathology in these patients; GPS was not associated with upgrading in surveillance biopsies and clinical variables (PSAD and percentage of positive biopsy cores) remained significantly associated with upgrading [78].

### 4.4. Decipher

Decipher is a genome classifier (GC) developed by GenomeDx Biosciences (Vancouver, BC, Canada). It is based on the analysis of the expression of 22 RNA biomarkers involved in multiple pathways related to the development and progression of PCa. This GC involves a complete transcriptome analysis of a specimen (obtained via prostatectomy, biopsy, or transurethral resection). The Decipher GC is currently considered to have the strongest level of scientific evidence among the available gene panels. Unlike other GCs, Decipher does not require any clinical data. Decipher was first validated in patients who underwent radical prostatectomy. The test results provide an estimate of the risk of developing metastatic progression in patients whose surgical specimen presents unfavourable characteristics (stage pT3 and/or positive margins) [79,80,81,82]. These initial findings were supported by subsequent studies that included postoperative radiotherapy (PORT) [83]. In that study of 139 patients treated with radical prostatectomy followed by PORT, Decipher predicted both biochemical recurrence and metastasis after post-prostatectomy radiotherapy.

In 2017, Spratt et al. [80] published the first meta-analysis to assess the performance of the Decipher test. That meta-analysis included data from five studies published from 2011–2016 involving 855 patients treated with radical prostatectomy. The patients were classified by Decipher into low (60.9%), intermediate (22.6%), or high risk (16.5%). At a median follow-up of eight years, the 10-year cumulative rate of metastasis by risk group was 5.5%, 15%, and 26.7% (*p* < 0.001), respectively. Decipher was an independent predictor of metastasis in all risk subgroups. The C-index for 10-year distant metastases for the clinical model alone was 0.76, rising to 0.81 when Decipher was included. This analysis confirmed the prognostic value of the Decipher score, which was independent from the standard clinicopathological variables (Gleason grade, pT stage, surgical margins, and PSA). The findings of that meta-analysis show that a low Decipher score is associated with long-term disease control after prostatectomy, regardless of when radiotherapy is initiated (i.e., ART vs. early SRT). By contrast, high to intermediate Decipher scores were associated with a worse prognosis; in these patient subgroups, PORT offers the greatest clinical benefit.

Other authors have also confirmed the value of the Decipher score to assess the expected impact of PORT. Dalela and colleagues [81] evaluated 512 patients with PCa who presented adverse pathological factors (stage pT3a, positive margins and/or positive nodes) after radical prostatectomy. At 10 years, patients with ≥2 poor prognostic factors who received PORT had a lower clinical recurrence rate than patients with <2 risk factors initially assigned to observation (10.1% vs. 42.1%, *P* = 0.012). Based on these findings, the authors concluded that this model reduced the risk of clinical recurrence in 25% of all patients with aggressive pathologic disease. The data provided by Decipher may improve decision-making regarding the indication for ART, potentially reducing overtreatment [84,85,86].

In the PRO-IMPACT study, Gore et al. [82] prospectively evaluated 265 patients who underwent radical prostatectomy to assess the influence of the Decipher score on the postoperative treatment decision (ART versus SRT). The initial treatment decision—ART in 150 and SRT in 115 patients—was based on clinicopathological criteria. The aim was to determine the impact of the Decipher test result on the clinical decision, which was changed in 18% and 32% of patients in the ART and SRT groups, respectively.

In a similar study, Marascio et al. [79] prospectively assessed the impact of Decipher in the postoperative setting in terms of clinical benefit (135 patients) and clinical utility (*n* = 3455). Clinical utility was quantified in terms of a change in the recommended treatment (39% of patients), with only three tests needed to change one treatment decision. In patients with a high risk Decipher score (61% of the sample) who received the recommended treatment (ART), the two-year biochemical recurrence rate was 3% versus 25% in patients who did not receive the recommended treatment, a finding that appears to confirm the clinical value of this test.

The RTOG 9601 study [87] found that androgen-deprivation therapy (ADT; bicalutamide 150 mg/d for two years) plus SRT improved overall survival (OS) at 12 years (HR, 0.77) while also reducing the risk of metastasis and cause-specific mortality in patients with clinical and pathological risk factors who underwent radical prostatectomy. However, not all patients benefit equally from hormonal treatment as outcomes depend on the PSA values prior to SRT. At the 2020 Genitourinary Cancers Symposium hosted by the American Society of Clinical Oncology (ASCO), the results of a post hoc analysis of 352 tissue specimens from the RTOG 9601 study were presented. These samples were evaluated to calculate the Decipher score, which was independently associated (as a continuous variable) with distant metastasis (HR, 1.19) and OS (HR, 1.16) after statistical adjustment for adverse pathological factors; this was the first clinical trial where treatment with ADT in patients with a low Decipher score had an estimated benefit on 12-year OS outcomes (2.4% versus 8.9% for patients treated with ADT with a high Decipher score), leading the authors to conclude that the Decipher test can improve decision-making to help prevent overtreatment with HT, a highly relevant finding given the adverse effects associated with hormonal therapy and its negative impact on quality of life.

There is substantial heterogeneity in the quality and extent of evidence across the various commercial biomarkers. No biomarker has strong evidence for the need for these tests currently in very low risk disease. Additional studies are warranted to demonstrate that these tests improve active surveillance in low-risk PCa. Post-operatively, the strongest evidence for the utility of Decipher testing is that it improves prognostication and discrimination: in a prospective trial and multiple prospective registries, Decipher has been proven to alter patient management. A post hoc analysis of a randomised phase III trial showed that Decipher can help to better select the patients likely to benefit from treatment intensification. Thus, as the data continue to diverge for each test, it may no longer be appropriate to view them as a uniform category, given their substantial differences in development, training, and validation.

Although many open questions remain, most of these are likely to be resolved in the coming years. Nevertheless, there is sufficient moderate-strength evidence for certain clinical scenarios to recommend the use of molecular biomarkers. In these cases, the results of gene assays, considered together with standard clinical and pathological factors, can substantially alter treatment selection; some examples of this would include the decision to recommend ART vs. early SRT, the advisability of concomitant ADT and radiotherapy, and the indication for active surveillance vs. radical treatment in well-selected cases [88] (Table 2). Future work is necessary to continue to refine how to best use these biomarkers in clinical practice.

## 5. Molecular Biomarkers in Advanced Prostate Cancer

Multiple therapeutic options have been shown to improve survival in patients with advanced prostate cancer. PCa is a highly heterogeneous disease [89,90,91], yet it is still managed as a single, homogenous disease, mainly due to the lack of validated biomarkers needed to correlate the molecular biology of the disease with the expected clinical course. Although the androgen receptor (AR) has long been a primary target of treatment, approximately 60% of patients present alterations in other molecular pathways, representing potentially-actionable novel treatment targets [91]. Numerous candidate biomarkers have been identified as potential prognostic or predictive indicators, which—if validated—could be used to guide treatment selection in clinical trials, potentially improving clinical outcomes in patients with advanced PCa.

### 5.1. DNA Repair Defects

In patients with metastatic castration-resistant PCa (mCRPC), approximately 25% of tumours present mutations in the genes involved in DNA repair, known as DDR genes [49,50,89,91,92,93,94]. From 12% to 16% of these are germline mutations [49,50]. In the studies published to date, BRCA2 is the most commonly mutated gene (both germline and somatic) [1,49,50,91,92]. The phase III PROFOUND trial included the largest number of prostate tumour specimens (*n* = 2792) screened for mutations in DDR-related genes. The findings of the PROFOUND trial demonstrated the efficacy of olaparib in the treatment of mCRPC in patients who failed to prior chemotherapy and androgen signalling inhibitor (ARSi) treatments [95]. Moreover, 28% of the tumour samples presented alterations in at least one of the 15 genes involved in the homologous recombination repair (HRR) pathway, with mutations detected both in primary tumours (27%) and metastatic lesions (32%). These data, considered together with the findings recently described by Mateo et al.—who found that DDR defects were present in a similar percentage of localised tumours (diagnostic biopsies) and in biopsied samples obtained from men (*n* = 61) with mCRPC [96]—confirm that alterations in the HRR pathway occur early in the course of disease in certain prostate tumours. Although HRR alterations are an early event, their prevalence is markedly higher in castration-resistant disease [49,50] compared to localised PCa [89,94], suggesting a correlation between HRR mutations and more aggressive forms of PCa.

In patients with mCRPC, the presence of DDR alterations has been identified as a biomarker of response to poly ADP-ribose polymerase (PARP) inhibitors [95,97,98,99,100,101], and to platinum-based chemotherapy [102,103,104]. Multiple clinical trials are currently underway to evaluate the efficacy of these drugs at different stages of the disease (Table 3). Nonetheless, the findings described above suggest that screening for these mutations could provide data that are valuable both for genetic counselling and to guide treatment selection.

The TOPARP-B trial evaluated the efficacy of olaparib in patients (*n* = 98) with mCRPC and DDR gene aberrations who had been previously treated with one or two taxane chemotherapy regimens. Patients were randomised to receive either 400 mg or 300 mg of olaparib twice daily, with a higher overall response rate (54.3% vs. 39.1%) in the higher dose group. However, due to treatment-related toxicity, 37% of patients in the 400 mg group required a dose reduction to 300 mg. Patients with BRCA1/2 mutations presented the best treatment response, with radiological and biochemical response rates of 52% and 77%, respectively, versus only 5% and 11.3% for other DDR gene mutations [97].

Preliminary results from the phase II TRITON2 [99] and GALAHAD trials [101] in patients with treatment-refractory mCRPC confirm the efficacy of two other PARP inhibitors, rucaparib and niraparib, respectively. Although both trials included patients with DDR defects, they used different gene panels and techniques to determine the presence of these defects. For example, the GALAHAD trial only enrolled patients with biallelic mutations. The preliminary data show that the highest objective response rate was obtained in patients with BRCA1/2 mutations, especially BRCA2. The data from these two trials suggest that response does not appear to be conditioned by the type of mutation, since response was similar among patients with somatic, germline, monoallelic, and biallelic mutations [99,101] a finding that was confirmed in the recent study by Jonsson et al. [105], who evaluated the role of BRCA1 and BRCA2 mutations in more than 17,000 patients (including 1042 with PCa). That study showed that, in certain tumours such as PCa, the clinical benefit of PARP inhibitors is similar, regardless of the type of mutation (germline, somatic, monoallelic, or biallelic).

A prospective analysis of 78 patients included in the TRITON2 trial with mutations in DDR genes other than BRCA1/2 showed that the presence of these alterations—particularly ATM, CDK12 and CHEK2—was associated with poor radiological and biochemical response rates (<11%) [100]. These results underscore the need for more studies to better elucidate how mutations in genes other than BRCA1/2 influences the activity of PARP inhibitors in PCa.

The phase III PROFOUND trial [95] was performed to evaluate patients with mCRPC and DDR mutations who had failed previous treatment with an ARSi (either abiraterone acetate or enzalutamide). Patients were randomised to receive olaparib 300 mg b.i.d. versus the ARSi that they had not previously received. Patients were stratified into two cohorts according to the type of gene mutations (Cohort A: BRCA1/2 and ATM vs. Cohort B: BARD1, BRIP1, CDK12, CHEK1, CHEK2, FANCL, PALB2, PPP2R2A, RAD51B, RAD51C, RAD51D, and RAD54L). In cohort A, radiological progression-free survival (the primary endpoint) was 7.4 months with olaparib versus 3.9 months for patients treated with abiraterone or enzalutamide (HR 0.34; 95% CI 0.25–0.47, *p* < 0.001). An exploratory analysis carried out to evaluate the individual effect of each gene found that BRCA2 seems to be the best predictor of response to olaparib. Importantly, 28% of the samples were, for various reasons, not suitable for sequencing [95] which is why alternative approaches, such as circulating DNA analysis, are being used in some studies with promising early results [106].

The presence of DDR gene alterations may have clinical implications not only for treatment with PARP inhibitors, but also for response to current treatments for mCRPC, such as taxanes or ARSi. The available retrospective evidence is controversial due to conflicting results [107,108,109]. PROREPAIR-B is the only prospective trial [50] to date to evaluate patients with mCRPC (*n* = 419) with germline DDR mutations (16% of the sample). In that study, the presence of a germline mutation in BRCA2 (gBRCA2) was confirmed as an independent prognostic factor of cause-specific survival (CSS). Moreover, the findings of that study also suggest that patients with gBRCA2 have worse CSS when treated with first-line taxanes followed by ARSi (10.7 vs. 28.4 months, *p* < 0.001), but not when the sequence is reversed (ARSi followed by taxanes: 24 vs. 31.3 months, *p* = 0.901). Those data, if confirmed, could position gBRCA2 as the first biomarker to guide first-line treatment selection in patients with mCRPC.

In other DNA repair pathways, such as DNA mismatch repair (MMR), the presence of a mutation (generally in MSH2, MSH6, MLH1, and PMS2) has been associated with microsatellite instability (MSI). Depending on the study, these alterations have been detected in 3%–12% of patients with mCRPC [49,50,91,109,110,111,112]. Abida et al. evaluated more than 1000 patients with PCa, finding that the most commonly altered gene was MSH2. Moreover, in 22% of patients with MMR alterations, the MSH2 mutation was present in the germline [112].

The presence of MSI is associated with genetically unstable tumours that have a tendency to accumulate mutations. This, in turn, leads to a higher burden of neoantigens, which may increase (hypothetically) the probability of response to immunotherapy [113]. In 2017, the Food and Drug Administration (FDA) approved pembrolizumab for the treatment of patients with alterations in this pathway (deficient-MMR or MSI), regardless of the tumour origin. However, a recent exploratory analysis of data from the phase II KEYNOTE-199 trial (performed to evaluate the role of pembrolizumab—an anti-programmed death receptor-1 (PD1)—in patients with mCRPC) was unable to find a clear association between the presence of alterations in DDR or MMR genes and response to pembrolizumab [114].

The phase III IMbassador250 trial (NCT 03016312) compared atezolizumab plus enzalutamide versus enzalutamide alone in patients with mCRPC. The trial, however, was discontinued early due to lack of efficacy, underscoring the need for more specific studies to determine the role of immunotherapy in advanced PCa.

### 5.2. PTEN Loss and PI3K/AKT Activation

Loss of PTEN is one of the most common molecular alterations in PCa, present in approximately 40% of patients with castration-resistant disease [90,115]. PTEN loss occurs early in the course of disease, with several studies showing a high correlation between PTEN loss in the primary tumour, metastatic lesions, plasma, and circulating tumour cells (CTC) [116,117]. Given that PTEN loss can occur through multiple mechanisms, including gene deletion, point mutations, or promoter methylation [118], the best technique to determine PTEN loss is quantification of protein expression by immunohistochemistry [119]. Loss of PTEN function leads to overactivation of the PI3K/AKT pathway, which is associated with increased AR signalling and worse prognosis [120,121]. This provides the rationale for the development of combined therapies in patients with mCRPC involving AR-targeted therapies and PI3K/AKT inhibitors.

A randomised phase II trial found that abiraterone plus ipatasertib (an AKT inhibitor) improves radiographic progression-free survival in patients with mCRPC and loss of PTEN [122]. Although these data are pending validation in a phase III trial that is currently underway (NCT03072238), the results demonstrate the clinical importance of molecular alterations as predictive biomarkers of response.

### 5.3. Androgen Receptor

Other common alterations observed in patients with PCa are those directly associated with AR signalling. Several studies have demonstrated the prognostic—and potential predictive—value of AR amplification, which is present in more than 50% of patients with castration-resistant PCa [123,124,125]. An exploratory analysis performed by Conteduca et al. found a lower risk of death for patients with AR amplification who received first-line docetaxel versus patients treated with ARSi, leading the authors to hypothesise that this alteration may be associated with resistance to ARSi, but not to taxanes in the first-line treatment of mCRPC [125]. Mutations in AR are common in castration-resistant disease, but not in early stage disease [90,91] The vast majority of these mutations appear after exposure to treatments that inhibit androgen signalling (T878A, F877L, among others), or after prolonged exposure to corticosteroid treatment (L702H) [116]. The presence of these alterations in liquid biopsy has been linked to resistance to treatments involved in androgen signalling, such as abiraterone and enzalutamide [123,124,126]. Similarly, numerous studies have found that the AR splice variant 7 (AR-V7) may also be important [127,128,129,130], as AR-V7 expression has been associated with worse clinical outcomes in patients treated with ARSi (both first- and second-line), but not with taxanes [127,128,129,130]. The PROPHECY trial (NCT02269982) is a prospective, multicentre study in patients with mCRCP treated with abiraterone/enzalutamide. That trial compared the two most important platforms for detecting AR-V7 in CTC: AR-V7 mRNA (AdnaTest) or AR-V7 nuclear localisation protein (Epic Sciences). The results of that trial showed that men with AR-V7–positive mCRPC had shorter PFS and OS outcomes, as well as fewer confirmed treatment responses (PSA and soft tissue) [131]. Although the prognostic value of this biomarker seems clear, its predictive value has not yet been clearly demonstrated and thus cannot be relied upon for clinical decision-making. Other alterations such as TP53 mutations have also been associated with poor clinical outcomes in mCRPC patients treated with ARSi [132,133].

In recent years, research into the biology of advanced PCa has helped us to better understand this disease. In this setting, several studies have identified the prognostic and/or predictive role of different biomarkers. For example, AR aberrations, including AR-V7, PTEN loss and/or TP53 mutations have all been associated with poor clinical outcomes in advanced PCa (prognostic biomarker). DDR alterations, particularly *BRCA2* mutations, have also been correlated with more aggressive disease; more importantly, recent studies have shown that patients with these alterations may benefit from PARP inhibitors and platinum-based chemotherapy (prognostic and predictive role). Ideally, in the near future, several of the biomarkers described here will be incorporated into routine clinical practice, thereby improving the clinical management of patients. Until then, more research is needed to better elucidate the molecular biology of PCa.

## 6. Conclusions

The molecular characterisation of PCa has become increasingly important to clinical decision-making in recent years. The unmet need to identify patients with clinically-insignificant PCa has prompted an intense search to find prognostic factors and molecular biomarkers that would allow us to minimise or prevent overdiagnosis and overtreatment in patients with localised disease. Fortunately, the increasing use of gene panels has improved patient stratification, thus allowing for more personalised therapies and follow-up schemes.

In the near future, optimisation of treatment sequencing, appropriate genetic counselling, and more data from the growing number of clinical trials that use genomic classification as inclusion criteria will, in all probability, increase the proportion of patients with identified mutations in DNA repair genes, potentially leading to the development of new biomarkers. A better understanding of PCa could change the initial management of the disease in most patients, creating an opportunity to apply new prognostic or predictive tools, although many of these still need to be validated before they can be incorporated into routine clinical practice.

## Figures and Tables

**Table 1:** Biomarkers for the diagnosis of prostate cancer before obtaining a positive biopsy. ncsPCa: non-clinically significant prostate cancer; csPCa: clinically significant prostate cancer; MP-MRI: Multiparametric-magnetic resonance imaging; fPSA: free PSA; tPSA: total PSA; iPSA: intact PSA; PCA3: Prostate cancer antigen 3.

Test	Specimen Type	Biomarker	Indication (Biopsy Setting)	Reduced Biopsies (%)	Reduced ncsPCa (%)	Missed csPCa (%)	Reduced MP-MRIs (%)
PHI (≥25)	Blood	(–2)pro-PSA/fPSA × √tPSA	Initial + Repeat	40	25	5	-
4KScore (≥9%)	Blood	tPSA, fPSA, iPSA, human kallikrein 2, clinical parameters	Initial + Repeat	43	ND	2.4	-
PCA3 (≥35)	Post-DRE urine	Ratio PCA3 mRNA/PSA mRNA × 1000	Repeat	67	ND	21	-
TMPRSS2-ERG (≥10)	Post-DRE urine	TMPRSS2-ERG expression	Repeat	-	-	-	-
MiPS (PCA3 (≥25) + TMPRSS2-ERG (≥10))	Post-DRE urine	TMPRSS2-ERG, PCA3, tPSA	Initial + Repeat	35	19	10	-
SelectMDX (≥2.8RS)	Post-DRE urine	HOXC6, DLX1, tPSA, clinical parameters	Initial + Repeat	42	ND	2	-
ExoDx (≥15,6)	Post-DRE urine	PCA3, exosomal ERG	Initial + Repeat	20	ND	7	-
ConfirmMDX	Biopsy prostate cores	GSTP1, APC, RASSF1	Repeat	-	-	-	-
MP-MRI	MP-MRI	T2, diffusion, contrast	Initial + Repeat	32	37/38	4/2	0
4KScore (≥7.5%)→MP-MRI	Blood→MP-MRI	4KScore + MP-MRI	Initial + Repeat	83	75	33	25
PCA3 (≥35)→MP-MRI	Post-DRE urine→MP-MRI	PCA3 + MP-MRI	Initial	76	87	48	52

**Table 2:** Tissue-based tests for localised prostate cancer prognosis and stratification.

Genomic Test	Reference	Tissue	Population (*n*)	Treatment	Outcome	Guidelines Recommendations ***
DECIPHER	Spratt DE et al. [70] 2018	Prostatectomy Biopsy	Training cohort (756) RP Validation cohort (235) ART	Radical Prostatectomy	DM→HiR/IR PCSM→HiR/IR	Post biopsy: NCCN very-LR/LR PCa in patients with ≥10 years life expectancy to define which could be candidates for AS versus definitive therapy Post-RP: 1) pT2 + positive margins; 2) pT3; 3) BF→To determine candidates for ART/SRT
				Adjuvant RT		
	Zhao SG et al. [74] 2016	Prostatectomy	Training cohort ART (196) Validation cohort (330) RP	Adjuvant RT	DM (10y)→HiP EBRT	
				Radical prostatectomy		
	Dalela D et al. [71] 2017	Prostatectomy	Adjuvant radiotherapy (112) Initial Observation (400) SRT if BF (168) *	Adjuvant RT	BF (10y)→GC SCORE	
	Kim HL et al. [75] 2019	Prostatectomy ** Biopsy	Radical Prostatectomy (266)	Active surveillance	AP→LR/IR	
	Berlin A et al. [76] 2019	Biopsy	Single Arm (121)	SRT +/− ADT	BF→GC SCORE 5y DM→GC SCORE	
ONCOTYPE	Eggener SE et al. [61] 2019	Prostatectomy Biopsy **	Initial AS (1200) Radical Prostatectomy (114)	Radical Prostatectomy (114)	Independent predictor of AP	Post-biopsy: NCCN very-LR/LR and favourable intermediate-risk PCa patients with ≥10 years life expectancy to define which could be candidates for AS versus definitive therapy
	Cullen J et al. [67] 2015	Biopsy	Single arm (431)	Radical Prostatectomy	BF→NCCN risk group/GPS DM→GPS/GS biopsy AP→GPS + GS, age, NCCN risk group	
PROLARIS	Freedland SJ et al. [63] 2013	Biopsy	Single arm (179)	EBRT +/− ADT	BF→CCP after EBRT/CF ** PCSM→CCP after EBRT	Post-biopsy: NCCN very-LR/LR and favourable intermediate-risk PCa in patients with ≥10 years life expectancy to define which could be candidates for AS versus definitive therapy
	Cuzick J et al. [59] 2011	Prostatectomy TURP	Single arm (410)	Radical Prostatectomy	BF PCSM→RP: CCP/ TURP: MVA CCP + PSA	
	Cuzick J et al. [78] 2015	Biopsy	Single arm 761	Active surveillance	PCSM→CCP+CAPRA	
	Cooperberg MR et al. [62] 2013	Prostatectomy	Single arm (413)	Radical Prostatectomy	BF→CCP + CAPRA	
	Klein EA et al. [66] 2014	Biopsy Prostatectomy	Biopsy (441) Prostatectomy (167) Validation cohort (395) ****	Radical Prostatectomy Active surveillance	Adverse pathology in RP High Stage/HiR biopsy→GPS	
PROMARK	Blume-Jensen P et al. [58] 2015	Biopsy	Training RP (381) Validation cohort (276)	Radical Prostatectomy Active surveillance	Adverse pathology in RP Gleason > 6	Post-biopsy: NCCN very-LR/LR PCa in patients with ≥10 year life expectancy to define which could be candidates for AS versus definitive therapy.

RP: Radical prostatectomy; ART: adjuvant radiotherapy; SRT: salvage radiotherapy; EBRT: external beam radiotherapy; AS: active surveillance; ADT: antiandrogen deprivation therapy; LR: low risk; IR: intermediate risk; HiR: high risk; GS: Gleason score; HiP: high PORTOS score; DM: distant metastasis; PCSM: prostate cancer specific mortality; BF: biochemical failure; GC ccore: RNA-based 22-gene genomic classifier (Decipher) score; AP: adverse pathology factors; GPS: genomic prostate score (oncotype); CCP: cell cycle progression score (Prolaris); GS + logPSA + T stage; CF **: clinical features: PSA, GS, biopsy positive cores + ADT; * patients treated with initial observation after RP but who experienced BF; ** prospective RP and retrospectively validated in the previous biopsies; *** NCCN Prostate Cancer Guidelines. v1.2020: Molecular Diagnostic Services Program (MolDX) Recommendation: Conditional, consider. EAU Guidelines: Molecular panels not routinely recommended; **** Validation cohort testing retrospectively collected needle biopsies from contemporary (1997–2011) patients with low to intermediate clinical risk.

**Table 3:** Ongoing clinical trials to evaluate poly ADP-ribose polymerase (PARP) inhibitors in patients with mCRPC.

PARP Inhibitor	Trial	Phase	Regimen	Patient Population
Rucaparib	TRITON2 (NCT02952534)	II	Rucaparib monotherapy	Post-abiraterone/enzalutamide and post-chemotherapy with DNA-repair abnormalities
	(NCT03442556)	II	Rucaparib	Patients who are responding after docetaxel + carboplatin with DNA-repair abnormalities
	TRITON3 (NCT02975934)	III	Rucaparib vs. abiraterone or enzalutamide or docetaxel	Patients with DNA-repair abnormalities (2L mCRPC)
Niraparib	BEDIVERE (NCT02924766)	I	Niraparib + apalutamide or abiraterone + prednisone	Post AR-targeted therapy and post-taxane
	QUEST (NCT03431350)	I/II	Niraparib + abiraterone or JNJ-63723283	Post AR-targeted therapy
	GALAHAD (NCT02854436)	II	Niraparib monotherapy	Post-chemotherapy with DNA-repair abnormalities
	MAGNITUDE (NCT03748641)	III	Niraparib + abiraterone vs. placebo + abiraterone	Patients with or without DNA-repair defects
Talazoparib	TALAPRO-1 (NCT03148795)	II	Talazoparib monotherapy	Post-abiraterone/enzalutamide and post-chemotherapy with DNA-repair abnormalities
	TALAPRO-2 (NCT03395197)	III	Talazoparib + enzalutamide vs. placebo + enzalutamide	First line mCRPC
Olaparib	(NCT01972217)	II	Olaparib + abiraterone vs. placebo + abiraterone	Post docetaxel mCRPC
	PROpel (NCT03732820)	III	Olaparib + abiraterone vs. placebo + abiraterone	First line mCRPC
	PROfound (NCT02987543)	III	Olaparib vs. abiraterone/enzalutamide	Post-abiraterone/enzalutamide mCRPC with HRR gene alterations
	KEYLINK-010 (NCT05834519)	III	Olaparib + pembrolizumab vs. abiraterone/enzalutamide	Post AR-targeted therapy and post-taxane
